# Training nursing professionals for immunobiological administration in the ventrogluteal region: an experience report

**DOI:** 10.1590/1980-220X-REEUSP-2026-0160en

**Published:** 2026-07-27

**Authors:** Gabriela Gonçalves Amaral, Júlia Falcão Bertoni, Stela de Azevedo Camtamos, Thayane Ingrid Xavier de Andrade, Juliana Ferreira da Silva, Maira de Castro Lima, Eliete Albano de Azevedo Guimarães, Valéria Conceição de Oliveira

**Affiliations:** 1 Universidade de São Paulo, Escola de Enfermagem, São Paulo, SP, Brazil.; 2 Universidade Federal de São João Del-Rei, Campus Centro-Oeste, Divinópolis, MG, Brazil.; 3 Universidade do Estado de Minas Gerais, Unidade Acadêmica Divinópolis, Divinópolis, MG, Brazil.; 4 Secretaria Municipal de Saúde, Central de Imunizações, Divinópolis, MG, Brazil.

**Keywords:** Primary Health Care, Professional Training, Nursing, Team, Vaccination, Immunization

## Abstract

**Objective:**

To report the experience of planning and developing a training program for immunobiological administration in the ventrogluteal region.

**Method:**

An experience report regarding a training program directed at nursing professionals, conducted in September 2024 through the integration of extension projects and in partnership with the Municipal Health Department of Divinópolis, Minas Gerais, Brazil.

**Results:**

Twenty-three training sessions were conducted, involving 278 professionals. Each session, lasting three hours, was structured into three stages: a theoretical approach to anatomy; a theoretical approach to immunobiological administration; and practical training in the technique for locating the ventrogluteal region. Activities were conducted by a faculty member, supported by a facilitator and at least two student monitors. The groups, composed of an average of 14 professionals, allowed individualized follow-up and greater interaction during discussions and practical activities.

**Conclusion:**

This report contributes by demonstrating the feasibility of implementing a theoretical-practical training program focused on immunobiological administration in the ventrogluteal region within the context of Primary Health Care, highlighting its potential to improve professional practice and increase safety in the vaccination process.

## INTRODUCTION

Vaccination constitutes a complex and constantly evolving field, with the nursing team being responsible for all stages of the process^(1)^. This complexity stems from frequent updates to vaccination schedules, the incorporation of new immunobiologicals, and the expansion of the age groups covered, requiring continuous training and systematic supervision of professionals working in vaccination rooms^(1,2,3)^.

This constant transformation does not always allow professionals to be adequately prepared to understand and communicate these changes, reinforcing the importance of well-structured training programs that combine theory with the practical application of vaccination^(4)^. Evidence indicates that structured training can significantly improve immunobiological administration practices, standardize procedures, and increase safety during the vaccination process^(5,6,7)^.

Although high-quality care should be grounded in up-to-date knowledge, there is often a lack of continuous qualification processes, resulting in doubts and difficulties in daily work routines and generating feelings such as fear, insecurity, and apprehension^(2)^. Evidence suggests that training initiatives have occurred sporadically and have been predominantly directed toward meeting emerging operational demands, reinforcing the need for investments in continuing education^(8)^. Therefore, the need to train nursing teams to perform all stages of the vaccination process is emphasized.

The Brazilian National Immunization Program schedule provides 51 immunobiologicals, including vaccines, sera, and immunoglobulins, ensuring protection for users of the public healthcare system throughout life^(9)^. Most of these immunobiologicals are administered intramuscularly, which reinforces the importance of adopting safe administration techniques. Inappropriate use of this route may result in complications such as abscesses, tissue irritation, neuropathies, and pain syndromes, making technical rigor in site selection and procedure execution essential^(10,11)^.

The deltoid muscle, vastus lateralis muscle, and the dorsogluteal and ventrogluteal regions are recommended sites for intramuscular administration. The dorsogluteal region is indicated for the administration of certain immunoglobulins but is considered the last option for immunobiologicals because of the greater risk of sciatic nerve injury and anatomical variations that may compromise the correct administration of the immunobiological^(11,12,13)^. In contrast, the ventrogluteal region is recommended as the first-choice site for adolescents and adults because it offers easy access and clear anatomical landmarks and is not located near major nerves and blood vessels, reducing the risk of injury and ensuring greater safety. It is also advantageous in reducing pain during administration^(11,14,15,16)^. Although anatomically advantageous and widely recommended, studies indicate that it remains underutilized due to a lack of knowledge of the technique, professional insecurity, and persistence in the use of traditional methods^(11,14,17)^.

Therefore, the need to train and update nursing professionals regarding immunobiological administration in the ventrogluteal region is evident, so that this site may be incorporated safely and effectively into the routine practice of vaccination rooms.

Accordingly, this study aimed to report the experience of planning and developing a training program for immunobiological administration in the ventrogluteal region.

## METHOD

### Study Design and Setting

This is an experience report, which enables the systematization, analysis, and critical reflection of practices developed within a professional context. This type of study allows the production of knowledge based on the authors’ experiences, contributing to the improvement of healthcare and nursing practices^(18)^. To assess the methodological rigor of the report, the Standards for Quality Improvement Reporting Excellence (SQUIRE 2.0) recommendations were adopted^(19)^.

The training program was conducted in September 2024 through the integration of the extension projects “*Educar para Vacinar*” (Educating to Vaccinate) and “*Conhecendo o Corpo Humano*” (Knowing the Human Body), from *Universidade Federal de São João del-Rei*, and the project “*Vacinação em Foco*” (Vaccination in Focus), from *Universidade do Estado de Minas Gerais*, in partnership with the Municipal Health Department of Divinópolis, a municipality located in the state of Minas Gerais, Southeastern Brazil.

### Experience Objective

To improve theoretical and practical knowledge regarding the technique of immunobiological administration in the ventrogluteal region, promoting safety, quality of care, and expanded use of an administration site recognized as safe and effective but still underutilized in the routine practice of vaccination rooms within the municipality.

### Experience Description

The training program was conducted in person using a theoretical-practical approach. It was developed based on a problem-posing, dialogical, and contextualized educational framework, respecting the participants’ social, cultural, and historical contexts^(20)^. Thus, the collective construction of knowledge was encouraged, strengthening the commitment to improving healthcare services and valuing the experiences of nursing professionals working in vaccination rooms within Primary Health Care units.

The training process was carefully planned to ensure content effectiveness and participant qualification. During the first meeting, an action plan was developed, including activity organization, schedule distribution, and the definition of responsibilities for each team member. It was established that each training session would last three hours and would encompass two main components: (1) the study of muscular and skeletal system anatomy; and (2) theoretical-practical content regarding immunobiological administration.

**Figure 1:**
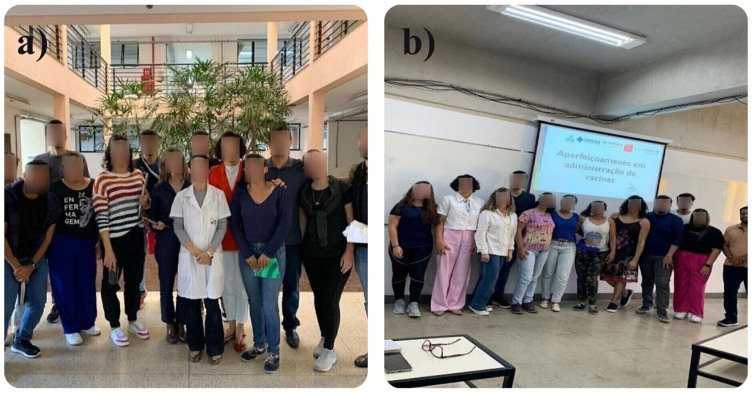
Preparation and pedagogical improvement phase of the team – Divinópolis, MG, Brazil, 2024.

As part of the preparation and pedagogical improvement phase, the team responsible for delivering the training participated in a workshop conducted by a faculty member from the “*Conhecendo o Corpo Humano*” (Knowing the Human Body) project, focusing on muscular and skeletal system anatomy. Particular emphasis was placed on anatomical landmarks that support the identification of immunobiological administration sites, including the deltoid muscle, vastus lateralis muscle, and the dorsogluteal and ventrogluteal regions (Figure 1A). Deepening anatomical knowledge was fundamental to strengthening the integration between theory and practice, fostering professionals who were more aware of their responsibilities and committed to improving the quality of care^(21)^.

In addition, to ensure uniformity and consistency in the content to be delivered, the individuals responsible for the training met beforehand to analyze discrepancies found in the scientific literature regarding immunobiological administration and to collaborate in the development of educational materials. This alignment process enabled the standardization of information and the adaptation of content to the most up-to-date and safest practices (Figure 1B).

It should be noted that the team adopted the ventrogluteal site location technique based on geometric landmarks, described in the literature as a reliable method for identifying the intramuscular administration site^(16)^. This technique uses palpable anatomical landmarks, such as the greater trochanter of the femur and the anterior superior iliac spine, forming an imaginary triangle along the iliac crest. The purpose of this technique is to reduce proximity to critical neural and vascular structures, thereby increasing the safety of immunobiological administration when compared with other administration sites^(12,16)^.

This experience report reflects the authors’ perceptions regarding the experience presented and discussed throughout the article. As it did not involve any form of data collection, submission to a Research Ethics Committee was not required. Furthermore, participants authorized the use of photographic records for scientific purposes through informed consent, and the anonymity of all individuals involved was ensured.

## RESULTS

Initially, all professionals working in the municipality’s vaccination rooms were invited to participate, including those assigned to extended-hours units, who perform their activities between 4:00 p.m. and 10:00 p.m. and generally do not participate in conventional training programs, as these are predominantly conducted during daytime hours. To this end, an invitation letter was sent to the technical managers of each Primary Health Care unit, containing the available dates and times. These professionals were responsible for coordinating with supervisors and organizing the participation of workers assigned to vaccination rooms.

In accordance with the schedule previously agreed upon with the Municipal Health Department of Divinópolis, 23 training sessions were conducted over two weeks in September 2024 (September 2–13, 2024). Activities were carried out during morning (n = 10), afternoon (n = 10), and evening (n = 3) shifts to enable the participation of the largest possible number of professionals from the municipality and partner institutions.

A total of 278 participants were trained, distributed as follows: nursing professionals working in the municipality’s vaccination rooms (n = 249); professionals from the municipality’s Special Immunobiologicals Reference Center (n = 5); representatives from partner higher education institutions (n = 13); professionals affiliated with the Municipal Health Department of a neighboring municipality that also partnered in the initiative (n = 11).

Each three-hour training session was organized into three stages: a theoretical approach to anatomy, a theoretical approach to the immunobiological administration process, and a practical component focused on performing the technique for locating the ventrogluteal administration site. Activities were conducted by a faculty member responsible for the session, supported by a facilitating faculty member and at least two student monitors, all previously trained for these roles. Each session included an average of 14 participants, enabling individualized follow-up and fostering interaction during discussions and practical activities.

### First Stage: Theoretical Approach to Anatomy

**Figure 2:**
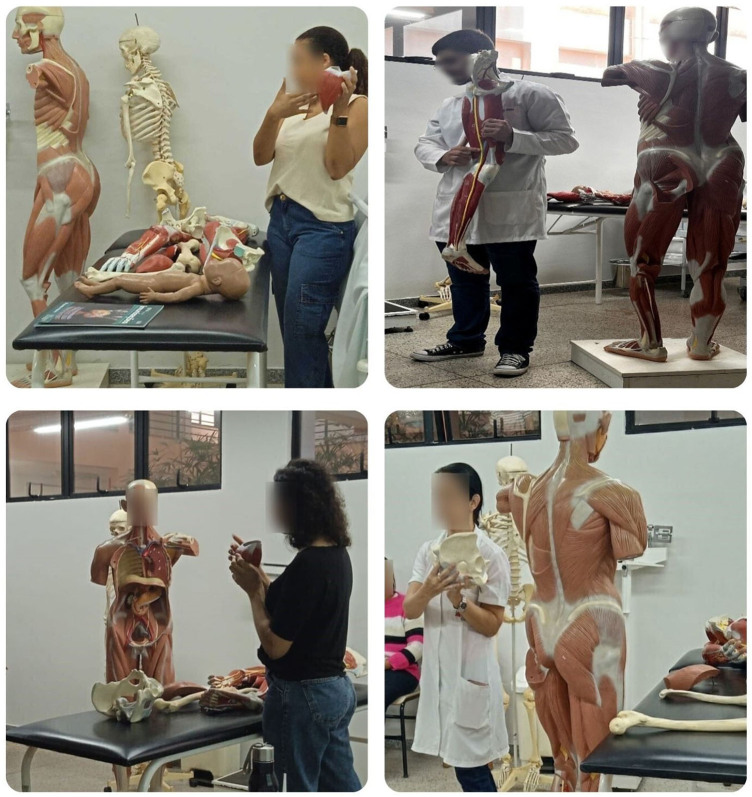
Theoretical approach to anatomy – Divinópolis, MG, Brazil, 2024.

This stage enabled participants to enhance their knowledge and clarify doubts regarding the anatomy of the muscles most frequently used in vaccination practice, while providing an in-depth study of the ventrogluteal region, the central focus of the training. To achieve this, anatomical models were used to facilitate the visualization and identification of anatomical structures. In the upper limb, the location of the deltoid muscle was explored; in the lower limb, the identification of the vastus lateralis muscle was addressed; and in the pelvic region, the observation of bony landmarks in conjunction with the femur was emphasized, as these structures are essential for locating the ventrogluteal region. Additionally, an anatomical human body model with removable gluteal muscles (gluteus maximus, gluteus medius, and gluteus minimus) was used, allowing precise visualization of the ventrogluteal region (Figure 2).

### Second Stage: Theoretical Approach to the Immunobiological Administration Process

**Figure 3:**
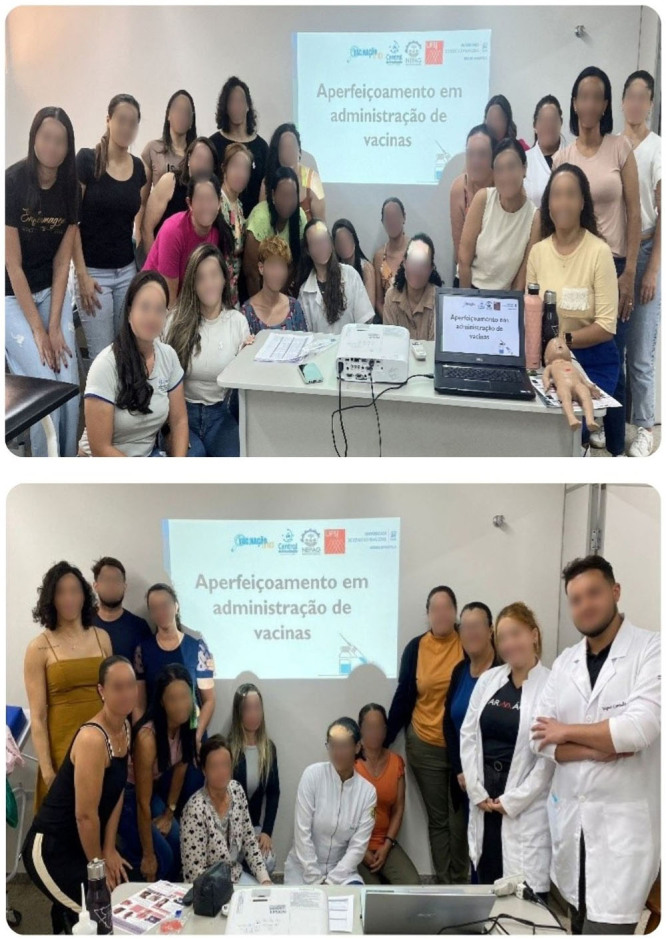
Theoretical approach to the immunobiological administration process – Divinópolis, MG, Brazil, 2024.

This stage was delivered through a lecture supported by digital resources and slide presentations. Using a participatory methodology, the lecture addressed essential topics, including: the concept of vaccination; required materials; precautions during vaccination; patient screening and guidance; assessment of muscle mass and selection of the administration site; routes of administration (oral, subcutaneous, and intramuscular); and procedures for waste disposal and hand hygiene (Figure 3).

Participants were encouraged to engage in collective discussions, which provided opportunities to clarify doubts, share experiences, and critically reflect on everyday practices within vaccination rooms. Discussions addressed perceptions regarding structural conditions, challenges encountered in daily work routines, and the relevance of training for improving the quality of care.

It was observed that one of the main challenges to implementing the technique of immunobiological administration in the ventrogluteal region is related to communication between the healthcare team and service users. Questions emerged regarding the most appropriate approaches for guiding users and explaining the benefits of using this administration site. As this anatomical region is still seldom used in the routine practice of vaccinators in the municipality and is considered “new” by many professionals, concerns were expressed about the possibility of causing surprise or rejection among the population.

Furthermore, a certain degree of insecurity among healthcare professionals regarding the performance of the technique was identified, a finding consistent with the literature^(11,14,17)^. Among younger professionals, the main difficulty reported was the lack of practical experience with the technique. Among more experienced professionals, resistance to change was highlighted, often associated with the consolidation of previously established practices and familiarity with traditionally used methods. Such resistance to change is also supported by international studies^(17,22)^. Moreover, one study demonstrated the low preference for the ventrogluteal region as the first choice for intramuscular administration, even among professionals with prior knowledge of the technique^(23)^.

Concerns were also expressed regarding the safety of using this region for administration. In this regard, evidence supports the safety of the ventrogluteal region^(11,14,15,16)^. A double-blind randomized clinical trial demonstrated that the aspiration-free intramuscular vaccination technique used in the hepatitis A vaccine administration was safe with respect to post-vaccination adverse events when compared with the conventional aspiration technique^(24)^. Moreover, a systematic review demonstrated that the ventrogluteal region presents a lower risk of iatrogenic nerve injury, as well as a lower occurrence of local and systemic side effects across different age groups^(25)^.

Reports of professional insecurity and lack of knowledge among the population reinforce the need for continuous educational actions and periodic incentives aimed at promoting the use of this anatomical region. Such initiatives should promote not only technical proficiency but also the strengthening of professionals’ communication skills, thereby facilitating team adherence and public acceptance of the technique. This need has also been reported in studies recommending training programs grounded in scientific evidence^(26,27)^.

In this context, with the aim of complementing educational materials and disseminating evidence-based information regarding immunobiological administration in the ventrogluteal region, an infographic was developed based on official Ministry of Health documents and anatomy literature (https://doi.org/10.17632/hfwkt3gznn.1)^(28)^. The primary objective of this resource was to facilitate understanding of immunobiological administration and the recommended anatomical sites. Through its accessible language and visual organization, the infographic contributed to knowledge acquisition, establishing itself as a relevant educational resource. It was subsequently printed in banner format and distributed to all vaccination rooms within the municipality’s Primary Health Care units.

The use of educational materials such as the infographic proved relevant for knowledge retention, as visual strategies and structured educational resources facilitate content consolidation and contribute to the standardization of care practices^(29)^. A quasi-experimental study conducted in Istanbul, Türkiye, with nursing students demonstrated that video-based instruction can improve competency in administering intramuscular injections in the ventrogluteal region, indicating the positive impact of educational strategies on practice qualification and procedural safety^(30)^.

The initiative also highlights that the use of supportive educational materials and institutional follow-up are facilitating factors for team adherence and greater public acceptance of the technique.

### Third Stage: Practical Approach

**Figure 4:**
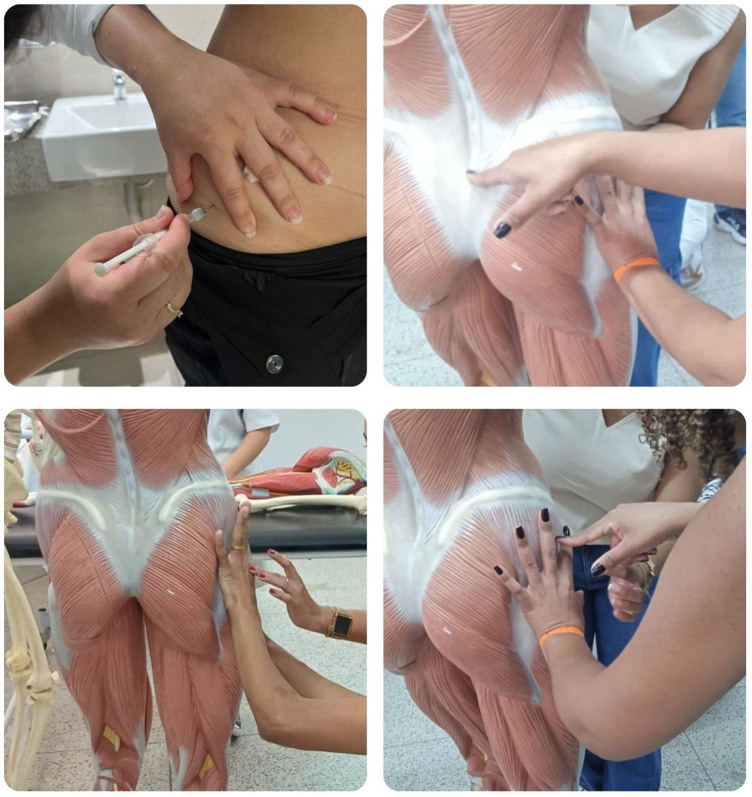
Practical approach to the immunobiological administration process – Divinópolis, MG, Brazil, 2024.

During this stage, participants were organized into pairs to practice locating the immunobiological administration site in the ventrogluteal region based on geometric landmarks^(12,16)^. Training included the recommended positioning for children, adolescents, adults, and older adults, using anatomical models and markers to identify the appropriate region. Participants who expressed interest were also given the opportunity to perform aseptic practice using syringes and needles, although no liquid administration was performed. For this activity, 3-mL syringes, 20 × 5.5 mm, 27 × 8 mm, and 25 × 7 mm needles, alcohol, cotton, and sharps disposal containers were provided (Figure 4).

During a single visit to a vaccination room, an individual may receive multiple vaccines, which often requires administration at different anatomical sites, particularly in cases of delayed vaccination schedules. Therefore, this experience reinforces the importance of structured continuing education as a strategic mechanism for incorporating evidence-based practices, especially when they involve changes to well-established routines.

Throughout the practical activities, participants received continuous guidance from the faculty member responsible and the student monitors, including individualized feedback regarding the accuracy of anatomical site identification and appropriate positioning for administration. In addition, strategies for approaching different user profiles were discussed, promoting the development of communication skills and confidence in performing the technique. This stage enabled the consolidation of theoretical knowledge, familiarization with the materials and instruments used, and the strengthening of participants’ confidence for future application of the technique in real-world situations.

The use of diversified educational resources proved essential for facilitating understanding of the correct anatomical sites and encouraging knowledge acquisition, thereby contributing to the improvement of the vaccination process. Furthermore, the training made it possible to identify important challenges, such as insecurity among less experienced professionals, resistance to change among more experienced professionals, and the need for effective communication strategies with users, particularly because this technique remains relatively unknown in the routine practice of vaccination rooms. Among the strategies adopted, special emphasis should be given to the infographic developed in banner format and made available in the municipality’s vaccination rooms, with the objective of supporting professional practice and facilitating clear and standardized communication with the population.

As limitations, it should be noted that the training sessions did not include an assessment of the perceptions or participation of the healthcare professionals involved, nor did they investigate the impact of the intervention on their daily practice. Future studies may advance this aspect through the use of validated instruments and systematic strategies to evaluate the impact of training on professional practice. Although no formal monitoring or evaluation instruments were applied, verbal reports and photographic records were subsequently received, indicating the incorporation of the technique in some units; however, these observations were not subjected to structured analysis within the scope of this report.

It should also be highlighted that, despite the relevance of the topic, there is a scarcity of recent studies and experience reports addressing the use of the ventrogluteal region for immunobiological administration, particularly within the Brazilian context. The available literature focuses predominantly on technical aspects of intramuscular administration, revealing gaps regarding implementation, acceptance, and professional training related to the use of the ventrogluteal region in the routine practice of vaccination rooms.

The Municipal Health Department continuously invests in updating and qualifying its professionals to ensure that they are fully prepared to safely manage the entire vaccination process, guaranteeing care, protection, and safety for the population. In a medium-sized municipality such as the one described in this report, which currently has 45 vaccination rooms within Primary Health Care and one Special Immunobiologicals Reference Center, continuing education on such a relevant topic as theoretical-practical training in immunobiological administration promoted the dissemination of knowledge and the development of participants’ skills. The training also enabled the identification and immediate correction of technical failures that may arise during routine work and compromise the quality of care provided to users. Consequently, this process contributes to the standardization of practices among vaccinators and has the potential to improve patient safety and service quality.

Finally, the importance of strengthening partnerships between educational institutions and healthcare services should be emphasized. Such partnerships encourage vaccinators’ commitment to seeking knowledge and, consequently, to improving the quality of vaccination techniques, resulting in greater competence in performing activities and enhanced safety in the daily routine of vaccination rooms throughout the municipality.

## CONCLUSION

This report contributes by demonstrating the feasibility of implementing a theoretical-practical training program focused on immunobiological administration in the ventrogluteal region within the context of Primary Health Care, highlighting its potential to improve professional practice and enhance safety in the vaccination process. By systematizing educational strategies that integrated expository teaching, participatory discussion, and practical simulation, the intervention promoted the development of technical competencies and strengthened professionals’ confidence in adopting the ventrogluteal region, an anatomical site that remains underutilized in the routine practice of vaccination rooms.

By expanding the discussion on the use of the ventrogluteal region for immunobiological administration, this report provides support for managers and professionals interested in improving patient safety and updating nursing practices in the field of vaccination.

## DATA AVAILABILITY

The supplementary material can be accessed at: https://doi.org/10.17632/hfwkt3gznn.1.
