# Supplementary material for: Training nursing professionals for immunobiological administration in the ventrogluteal region: an experience report
Source: Rev Esc Enferm USP. 2026 Jul 27;60:e20260160. doi: 10.1590/1980-220X-REEUSP-2026-0160en (PMC13411131; doi:10.1590/1980-220X-REEUSP-2026-0160en)
Supplement: Translation

# Translation: Capacitação de profissionais de enfermagem para administração de imunobiológicos na região ventroglútea: relato de experiência

**DOI:** 10.1590/1980-220X-REEUSP-2026-0160pt

**Authors:** Gabriela Gonçalves Amaral, Júlia Falcão Bertoni, Stela de Azevedo Camtamos, Thayane Ingrid Xavier de Andrade, Juliana Ferreira da Silva, Maira de Castro Lima, Eliete Albano de Azevedo Guimarães, Valéria Conceição de Oliveira

**Affiliations:** 1 Universidade de São Paulo, Escola de Enfermagem, São Paulo, SP, Brasil.; 2 Universidade Federal de São João Del-Rei, Campus Centro-Oeste, Divinópolis, MG, Brasil.; 3 Universidade do Estado de Minas Gerais, Unidade Acadêmica Divinópolis, Divinópolis, MG, Brasil.; 4 Secretaria Municipal de Saúde, Central de Imunizações, Divinópolis, MG, Brasil.

**Keywords:** Atenção Primária à Saúde, Capacitação Profissional, Equipe de Enfermagem, Vacinação, Imunização

## Abstract

**Objetivo:**

Relatar a experiência de planejamento e desenvolvimento de capacitação para administração de imunobiológico na região ventroglútea.

**Método:**

Relato de experiência, referente à capacitação dirigida a profissionais de enfermagem, realizada em setembro de 2024, com integração de projetos de extensão e parceria com a Secretaria Municipal de Saúde de Divinópolis, Minas Gerais.

**Resultados:**

Realizaram-se 23 capacitações, com a participação de 278 profissionais. Cada encontro, com duração de três horas, foi estruturado em três momentos: abordagem teórica sobre anatomia; teórica sobre administração de imunobiológicos; e prática da técnica de localização da região ventroglútea. As atividades foram conduzidas por um professor, com apoio de um mediador e ao menos dois monitores. As turmas, compostas em média por 14 profissionais, permitiram acompanhamento individualizado e maior interação nas discussões e práticas.

**Conclusão:**

Este relato contribui ao demonstrar a viabilidade da implementação de uma capacitação teórico-prática voltada à administração de imunobiológicos na região ventroglútea no contexto da Atenção Primária à Saúde, evidenciando potencial para qualificar a prática profissional e ampliar a segurança no processo de vacinação.

## INTRODUÇÃO

A vacinação constitui um campo complexo e em constante evolução, cabendo à equipe de enfermagem a responsabilidade por todas as etapas do processo^(1)^. Essa complexidade decorre das frequentes atualizações nos calendários vacinais, da incorporação de novos imunobiológicos e da ampliação das faixas etárias contempladas, o que exige capacitação contínua e supervisão sistemática dos profissionais que atuam nas salas de vacinação^(1,2,3)^.

Essa constante transformação nem sempre permite que os profissionais estejam preparados para compreendê-las e compartilhá-las adequadamente, o que reforça a importância de capacitações bem estruturadas, que aliem a teoria à aplicação prática da vacinação^(4)^. Evidências indicam que treinamentos estruturados podem melhorar significativamente as práticas de administração de imunobiológicos, padronizar procedimentos e aumentar a segurança durante o procedimento de vacinação^(5,6,7)^.

Embora uma assistência de qualidade deva estar fundamentada em conhecimentos atualizados, observa-se, muitas vezes, a ausência de processos de qualificação contínua, o que resulta em dúvidas e dificuldades no cotidiano de trabalho, despertando sentimentos como medo, insegurança e receio^(2)^. Evidências apontam que as capacitações têm ocorrido de forma pontual e predominantemente direcionadas à operacionalização de demandas emergentes, reforçando a necessidade de investimentos em educação continuada^(8)^. Reforça-se, portanto, a necessidade de capacitação da equipe de enfermagem para atuação em todas as fases do processo de vacinação.

O calendário vacinal do Programa Nacional de Imunizações brasileiro disponibiliza 51 imunobiológicos, como vacinas, soros e imunoglobulinas, garantindo proteção aos usuários da rede pública de saúde ao longo da vida^(9)^. A maioria desses imunobiológicos é administrada por via intramuscular, o que reforça a importância de adotar técnicas seguras de administração. A utilização inadequada dessa via pode acarretar agravos ao paciente, como abscessos, irritações teciduais, neuropatias e algias, tornando imprescindível o rigor técnico na definição da região de aplicação e na execução do procedimento^(10,11)^.

Os músculos deltoide, vasto lateral da coxa, bem como as regiões dorso e ventroglútea, são recomendados para a administração intramuscular. A região dorsoglútea é indicada para a administração de determinadas imunoglobulinas, mas é considerada a última opção para imunobiológicos, devido ao maior risco de lesão do nervo ciático e às variações anatômicas que podem comprometer a correta aplicação do imunobiológico^(11,12,13)^. Já a região ventroglútea é indicada como primeira opção na administração em adolescentes e adultos, por apresentar facilidade de acesso e delimitação anatômica, além de não estar próxima a nervos e vasos importantes, reduzindo o risco de lesões e garantindo maior segurança, sendo também vantajosa na redução da dor durante a administração^(11,14,15,16)^. Embora anatomicamente vantajosa e amplamente recomendada, estudos apontam sua subutilização associada ao desconhecimento da técnica, à insegurança profissional e à persistência no uso de métodos tradicionais^(11,14,17)^.

Portanto, evidencia-se a necessidade de capacitar e atualizar os profissionais de enfermagem quanto à administração dos imunobiológicos na região ventroglútea, de modo a incorporá-la de forma segura e eficaz na rotina das salas de vacinação.

Nesse sentido, o objetivo do estudo é relatar a experiência de planejamento e desenvolvimento de capacitação para administração de imunobiológico na região ventroglútea.

## MÉTODO

### Tipo e Local do Estudo

Trata-se de relato de experiência, que possibilita a sistematização, a análise e a reflexão crítica sobre práticas desenvolvidas no contexto profissional. Esse tipo de estudo permite a produção de conhecimento a partir da vivência dos autores, contribuindo para a qualificação das práticas em saúde e enfermagem^(18)^. Para a análise do rigor metodológico do relato, foram utilizadas as recomendações do *Standards for QUality Improvement Reporting Excellence* (SQUIRE 2.0)^(19)^.

A capacitação foi realizada em setembro de 2024, por meio da integração dos projetos de extensão “Educar para Vacinar” e “Conhecendo o Corpo Humano”, da Universidade Federal de São João del-Rei, e do projeto “Vacinação em Foco”, da Universidade do Estado de Minas Gerais, em parceria com a Secretaria Municipal de Saúde de Divinópolis, município do estado de Minas Gerais, na região Sudeste do Brasil.

### Objetivo da Experiência

Aperfeiçoar o conhecimento teórico e prático sobre a técnica de administração de imunobiológico na região ventroglútea, promovendo a segurança, a qualificação do cuidado e a ampliação do uso de um sítio de aplicação reconhecido como seguro e eficaz, mas ainda subutilizado na prática cotidiana das salas de vacinação do respectivo município.

### Descrição da Experiência

 A capacitação foi desenvolvida presencialmente com enfoque teórico-prático. Foi conduzida com base em uma abordagem problematizadora, dialógica e contextualizada, respeitando os contextos sociais, culturais e históricos dos participantes^(20)^. Assim, promoveu-se a construção coletiva do conhecimento, fortalecendo o compromisso com a melhoria dos serviços de saúde e a valorização das vivências dos profissionais de enfermagem atuantes das salas de vacinação das unidades de Atenção Primária à Saúde.

O processo de capacitação foi cuidadosamente planejado para garantir a eficácia do conteúdo e a qualificação dos participantes. No primeiro encontro, elaborou-se o plano de ação, com a organização das atividades, a distribuição de horários e a definição das funções de cada membro da equipe. Estabeleceu-se que cada sessão de capacitação teria duração de três horas, contemplando dois eixos principais: (1) o estudo da anatomia dos sistemas muscular e esquelético; e (2) conteúdos teórico-práticos sobre a administração de imunobiológicos.

Como parte da etapa de preparação e aprimoramento pedagógico, a equipe responsável por ministrar a capacitação participou de um treinamento com uma docente do projeto “Conhecendo o Corpo Humano”, cuja temática abordou a anatomia do sistema muscular e esquelético. O foco esteve nos pontos de referência anatômicos, que auxiliam na identificação dos locais de aplicação dos imunobiológicos, incluindo os músculos deltoide, vasto lateral da coxa e região dorso e ventroglútea (Figura 1A). O aprofundamento sobre o conhecimento anatômico foi fundamental para ampliar a integração entre teoria e prática, formando profissionais mais conscientes de suas responsabilidades e comprometidos com a melhoria da qualidade da assistência^(21)^.

**Figura 1:** Etapa de preparação e aprimoramento pedagógico da equipe – Divinópolis, MG, Brasil, 2024.

Além disso, visando assegurar a uniformidade e a consistência do conteúdo a ser ministrado, os responsáveis pela capacitação reuniram-se previamente para analisar divergências encontradas na literatura científica sobre administração de imunobiológicos e para colaborar na construção dos materiais didáticos. Esse momento de alinhamento permitiu a padronização das informações e a adequação dos conteúdos às práticas mais atualizadas e seguras (Figura 1B).

Destaca-se que a equipe adotou a técnica de localização da região ventroglútea baseada em referências geométricas, descrita na literatura como método confiável para identificar o sítio de administração intramuscular^(16)^. Para tal, utilizam-se pontos anatômicos palpáveis, como o trocânter maior do fêmur e a espinha ilíaca ântero-superior, formando um triângulo imaginário ao longo da crista ilíaca. Essa técnica visa reduzir a proximidade de estruturas nervosas e vasculares críticas, o que pode favorecer maior segurança na administração de imunobiológicos em comparação com outros sítios de aplicação^(12,16)^.

Este relato de experiência reflete as percepções das autoras acerca da vivência apresentada e discutida ao longo do artigo. Por não envolver qualquer forma de coleta de dados, não foi necessária a submissão ao Comitê de Ética em Pesquisa. Ademais, os participantes autorizaram o uso dos registros fotográficos para fins científicos, mediante consentimento, sendo assegurada a não identificação dos envolvidos.

## RESULTADOS

Inicialmente, todos os profissionais da rede que atuavam nas salas de vacinação do município foram convidados a participar, incluindo aqueles vinculados às unidades com horário estendido, que desempenham suas atividades no período das 16h às 22h e, em geral, não participam das capacitações convencionais, por ocorrerem predominantemente no período diurno. Para tanto, foi encaminhada carta-convite aos responsáveis técnicos de cada unidade de Atenção Primária à Saúde contendo as opções de datas e horários disponíveis. Esses profissionais ficaram responsáveis pelo alinhamento com os supervisores e pela organização da participação dos trabalhadores atuantes nas salas de vacinação.

Em conformidade com o cronograma previamente pactuado com a Secretaria Municipal de Saúde de Divinópolis, foram realizadas 23 capacitações ao longo de duas semanas do mês de setembro de 2024 (02/09/2024 a 13/09/2024). As atividades ocorreram nos turnos matutino (n = 10), vespertino (n = 10) e noturno (n = 3), de modo a possibilitar a participação do maior número possível de profissionais do município e instituições parceiras.

Foram capacitados 278 participantes, distribuídos da seguinte forma: profissionais de enfermagem das salas de vacinação do município (n = 249); profissionais do Centro de Referência para Imunobiológicos Especiais do município (n = 5); representantes de instituições de ensino superior parceiras (n = 13); e profissionais vinculados à Secretaria Municipal de Saúde de um município vizinho, também parceiro da iniciativa (n = 11).

Cada capacitação, que teve duração de três horas, foi organizada em três momentos: abordagem teórica sobre anatomia, abordagem teórica sobre processo de administração de imunobiológicos e abordagem prática, que contemplaram a execução da técnica de localização do local de administração de imunobiológicos na região ventroglútea. As atividades foram conduzidas por um professor responsável, com o apoio de um professor mediador e, no mínimo, dois estudantes monitores, todos previamente preparados para desempenhar essas funções. Em cada sessão de capacitação, participaram, em média, 14 participantes, o que possibilitou um acompanhamento individualizado e favoreceu a interação durante as discussões e as atividades práticas.

### Primeiro Momento: Abordagem Teórica Sobre Anatomia

Esse momento possibilitou o aprimoramento dos conhecimentos e o esclarecimento de dúvidas acerca da anatomia dos músculos mais utilizados na prática vacinal, além de aprofundar o estudo da região ventroglútea, foco central da capacitação. Para tanto, foram utilizadas peças anatômicas que favoreceram a visualização e a delimitação das estruturas. No membro superior, explorou-se a localização do músculo deltoide; no membro inferior, explorou-se a identificação do vasto lateral da coxa; e na pelve, explorou-se a observação das referências ósseas em conjunto com o fêmur, estruturas importantes para a localização da região ventroglútea. Ademais, recorreu-se a uma peça anatômica do corpo humano com estruturas removíveis dos músculos glúteos (máximo, médio e mínimo), o que possibilitou a visualização precisa da região ventroglútea (Figura 2).

**Figura 2:** Abordagem teórica sobre anatomia – Divinópolis, MG, Brasil, 2024.

### Segundo Momento: Abordagem Teórica Sobre Processo de Administração de Imunobiológicos

Esse momento foi mediado por uma aula apoiada por recursos digitais, com apresentação em formato de *slides*. Com metodologia participativa, a aula abordou tópicos essenciais, como: conceito de vacinação; materiais necessários; cuidados na vacinação; triagem e orientação ao usuário; avaliação da musculatura e definição do local de aplicação; vias de administração (oral, subcutânea e intramuscular); e cuidados com o descarte de resíduos e a higienização das mãos (Figura 3).

**Figura 3:** Abordagem teórica sobre processo de administração de imunobiológicos – Divinópolis, MG, Brasil, 2024.

Os participantes foram incentivados a participar dos debates coletivos, que possibilitaram o esclarecimento de dúvidas, o compartilhamento de experiências e a reflexão crítica acerca do cotidiano nas salas de vacinação. As discussões contemplaram percepções sobre as condições estruturais, os desafios enfrentados na rotina de trabalho e a relevância da capacitação para a qualificação da prática do cuidado.

Verificou-se que um dos principais desafios para a implementação da técnica de administração de imunobiológicos na região ventroglútea está relacionado à comunicação entre a equipe de saúde e os usuários do serviço. Surgiram dúvidas quanto às abordagens mais adequadas para orientar os usuários e esclarecer os benefícios do uso desse local de administração. Por tratar-se de uma região corporal ainda pouco explorada na prática cotidiana dos vacinadores do município e, para muitos, considerada “nova”, manifestou-se a preocupação quanto à possibilidade de causar estranhamento ou rejeição por parte da população.

Ademais, identificou-se certa insegurança entre os profissionais de saúde na realização da técnica, achado corroborado pela literatura^(11,14,17)^. Entre os mais jovens, a principal dificuldade referida foi a falta de experiência prática com a técnica. Já entre os mais experientes, destacou-se a resistência à mudança, frequentemente associada à consolidação de práticas previamente estabelecidas e à familiaridade com métodos tradicionalmente utilizados. Tal resistência à mudança é corroborada com estudos internacional^(17,22)^. Além disso, estudo evidenciou a baixa escolha da região ventroglútea como primeira opção para administração intramuscular, mesmo entre profissionais com conhecimento prévio sobre a técnica^(23)^.

Evidenciou-se, também, entre os profissionais receio quanto à segurança da utilização dessa região para a aplicação da técnica. Nesse sentido, evidências atestam a segurança dessa região^(11,14,15,16)^. Ensaio clínico randomizado duplo-cego evidenciou que a técnica de vacinação intramuscular sem aspiração, utilizada na administração da vacina contra hepatite A, mostrou-se segura em relação aos eventos adversos pós-vacinação quando comparada à técnica convencional com aspiração^(24)^. Além disso, revisão sistemática demonstrou que a região apresenta menor risco de lesão nervosa iatrogênica, bem como menor ocorrência de efeitos colaterais locais e sistêmicos em diferentes faixas etárias^(25)^.

Os relatos de insegurança do profissional e de desconhecimento por parte da população reforçam a necessidade de ações educativas contínuas e de incentivos periódicos voltados à utilização dessa região anatômica. Essas ações devem promover não apenas o domínio técnico, mas também o fortalecimento das competências comunicativas dos profissionais, de modo a favorecer a adesão da equipe e a aceitação da população em relação à técnica. Tal necessidade também é relatada em estudos que sugerem a realização de treinamentos fundamentados em evidências científicas^(26,27)^.

Nesse contexto, visando complementar os materiais didáticos e disseminar informações sobre a administração de imunobiológicos na região ventroglútea, baseado em evidências científicas, foi elaborado um infográfico fundamentado em documentos oficiais do Ministério da Saúde e em literatura de anatomia (https://doi.org/10.17632/hfwkt3gznn.1)^(28)^. O principal objetivo do recurso foi facilitar a compreensão sobre a administração de imunobiológicos e os locais anatômicos recomendados. Pela linguagem acessível e pela organização visual, o infográfico contribuiu para a assimilação do conteúdo, configurando-se como um recurso pedagógico relevante, posteriormente impresso em tamanho de *banner* e distribuído a todas as salas de vacinação das unidades de Atenção Primária à Saúde do município.

O uso de materiais educativos, como o infográfico desenvolvido, mostrou-se relevante para a fixação do conhecimento, pois estratégias visuais e recursos didáticos estruturados favorecem a consolidação do conteúdo e auxiliam na padronização das práticas assistenciais^(29)^. Estudo quase-experimental, realizado em Istambul, Turquia, com estudantes de enfermagem, demonstrou que a instrução por meio de vídeo pode melhorar a competência na administração de injeções intramusculares na região ventroglútea, indicando impacto positivo de estratégias educacionais na qualificação da prática e na segurança da técnica^(30)^.

A iniciativa ressalta, ainda, que o uso de materiais educativos de apoio e o acompanhamento institucional configuram elementos facilitadores para a adesão da equipe e maior aceitabilidade da técnica pela população.

### Terceiro Momento: Abordagem Prática

Nesse momento, os participantes foram organizados em duplas para treinar a localização do local de administração de imunobiológicos na região ventroglútea, com base em referências geométricas^(12,16)^. O treinamento contemplou os posicionamentos recomendados para crianças, adolescentes, adultos e idosos, utilizando-se peças anatômicas e canetas para a marcação da região adequada. Aos que manifestaram interesse, foi oportunizada a prática asséptica com seringas e agulhas sem, contudo, realizar a administração de líquidos. Para essa atividade, foram disponibilizadas seringas de 3 ml, agulhas de calibres 20 × 5,5 mm, 27 × 8 mm e 25 × 7 mm, além de álcool, algodão e caixa coletora para materiais perfurocortantes (Figura 4).

**Figura 4:** Abordagem teórica sobre o processo de administração de imunobiológicos – Divinópolis, MG, Brasil, 2024.

Durante uma única visita à sala de vacinação, o indivíduo pode receber múltiplas vacinas, o que exige, especialmente em casos de atraso vacinal, a aplicação em diferentes sítios anatômicos. Assim, a experiência reforça a importância da educação permanente estruturada como dispositivo estratégico para incorporação de práticas baseadas em evidências, especialmente quando envolvem mudanças em rotinas consolidadas.

Durante a prática, os participantes foram constantemente orientados pelo professor responsável e pelos monitores, recebendo *feedback* individual sobre a precisão da localização anatômica e a postura adequada para a administração. Além disso, foram discutidas estratégias para abordar diferentes perfis de usuários, promovendo o desenvolvimento de competências comunicativas e a segurança na execução da técnica. Essa etapa permitiu a consolidação dos conhecimentos teóricos, a familiarização com os materiais e instrumentos utilizados, bem como o fortalecimento da confiança dos participantes para a futura aplicação da técnica em situações reais.

A utilização de recursos didáticos diversificados mostrou-se fundamental para facilitar a compreensão dos locais anatômicos corretos e incentivar a assimilação do conteúdo, contribuindo para a qualificação do processo de vacinação. Ademais, a capacitação permitiu identificar desafios importantes, como a insegurança entre profissionais menos experientes, a resistência à mudança entre os mais experientes e a necessidade de estratégias de comunicação eficazes com os usuários, especialmente por se tratar de uma técnica ainda pouco conhecida na rotina das salas de vacinação. Entre as estratégias adotadas, destaca-se a disponibilização do infográfico construído em formato de banner, disponibilizado nas salas de vacinação do município, com o objetivo de apoiar a prática profissional e favorecer a comunicação clara e padronizada com a população.

Como limitações, ressalta-se que os momentos não incluíram a avaliação da percepção e da participação dos profissionais de saúde envolvidos na capacitação, nem investigaram os impactos da intervenção em sua prática cotidiana. Enfatiza-se que pesquisas futuras poderão avançar nesse aspecto, mediante utilização de instrumentos validados e estratégias sistemáticas de avaliação do impacto da capacitação na prática profissional. Embora não tenham sido aplicados instrumentos formais de acompanhamento ou avaliação da capacitação, foram posteriormente recebidos relatos verbais e registros fotográficos que evidenciaram a incorporação da técnica em algumas unidades, os quais, contudo, não foram objeto de análise estruturada no âmbito deste relato.

Destaca-se também que, apesar da relevância da temática, observa-se escassez de estudos recentes e de relatos de experiência que abordem a utilização da região ventroglútea para administração de imunobiológicos, especialmente no contexto nacional. A literatura disponível concentra-se, predominantemente, em aspectos técnicos da administração intramuscular, evidenciando lacunas quanto à implementação, à aceitação e à capacitação profissional voltadas ao uso da região ventroglútea na prática cotidiana das salas de vacinação.

A Secretaria Municipal de Saúde do município investe continuamente na atualização e na qualificação de seus profissionais para que estejam plenamente preparados para atuar no manejo seguro de todo o processo de vacinação, garantindo acolhimento, proteção e segurança à população. Em um município de médio porte, como o deste relato, que conta atualmente com 45 salas de vacinação na Atenção Primária à Saúde e um Centro de Referência para Imunobiológicos Especiais, a educação permanente sobre um tema de tamanha relevância, como o treinamento teórico-prático em administração de imunobiológicos, favoreceu a disseminação do conhecimento e o desenvolvimento de habilidades dos participantes. O treinamento também possibilitou a identificação e a correção imediata de falhas técnicas que podem surgir na rotina do trabalho e comprometer a qualidade da assistência aos usuários. Dessa forma, esse processo contribui para a padronização das condutas entre os vacinadores, com potencial para favorecer a segurança do paciente e a qualidade do serviço prestado.

Por fim, ressalta-se a importância de se consolidar a parceria das instituições de ensino com os serviços de saúde, estimulando o comprometimento dos vacinadores com a busca pelo conhecimento e, consequentemente, com a busca pela qualidade nas técnicas de vacinação, o que reflete em maior competência na execução das atividades e maior segurança no cotidiano das salas de vacinação no município.

## CONCLUSÃO

Este relato contribui ao demonstrar a viabilidade de implementação de uma capacitação teórico-prática voltada à administração de imunobiológicos na região ventroglútea no contexto da Atenção Primária à Saúde, evidenciando potencial para qualificar a prática profissional e ampliar a segurança no processo de vacinação. Ao sistematizar estratégias educativas que integraram abordagem expositiva, discussão participativa e simulação prática, a intervenção favoreceu o desenvolvimento de competências técnicas e o fortalecimento da confiança dos profissionais para adoção da região ventroglútea, sítio anatômico ainda pouco explorado na rotina das salas de vacinação.

Ao ampliar o debate sobre a utilização da região ventroglútea na administração de imunobiológicos, o relato oferece subsídios para gestores e profissionais interessados na qualificação da segurança assistencial e na atualização das práticas de enfermagem no âmbito da vacinação.

## DISPONIBILIDADE DE DADOS

O material suplementar pode ser consultado em: https://doi.org/10.17632/hfwkt3gznn.1.

# Translation: Capacitación de profesionales de enfermería para la administración de productos inmunobiológicos en la región ventroglútea: informe de experiencia

**DOI:** 10.1590/1980-220X-REEUSP-2026-0160es

**Authors:** Gabriela Gonçalves Amaral, Júlia Falcão Bertoni, Stela de Azevedo Camtamos, Thayane Ingrid Xavier de Andrade, Juliana Ferreira da Silva, Maira de Castro Lima, Eliete Albano de Azevedo Guimarães, Valéria Conceição de Oliveira

**Affiliations:** 1 Universidade de São Paulo, Escola de Enfermagem, São Paulo, SP, Brasil.; 2 Universidade Federal de São João Del-Rei, Campus Centro-Oeste, Divinópolis, MG, Brasil.; 3 Universidade do Estado de Minas Gerais, Unidade Acadêmica Divinópolis, Divinópolis, MG, Brasil.; 4 Secretaria Municipal de Saúde, Central de Imunizações, Divinópolis, MG, Brasil.

**Keywords:** Atención Primaria de Salud, Capacitación Profesional, Grupo de Enfermería, Vacunación, Inmunización

## Abstract

**Objetivo:**

Relatar la experiencia de planificación y desarrollo de una capacitación para la administración de inmunobiológicos en la región ventroglútea.

**Método:**

Relato de experiencia, referente a la capacitación dirigida a profesionales de enfermería, realizada en septiembre de 2024, con integración de proyectos de extensión y en colaboración con la Secretaría Municipal de Salud de Divinópolis, *Minas Gerais*.

**Resultados:**

Se llevaron a cabo 23 sesiones de capacitación, con la participación de 278 profesionales. Cada sesión, de tres horas de duración, se estructuró en tres partes: enfoque teórico sobre anatomía; teórico sobre la administración de inmunobiológicos; y práctica de la técnica de localización de la región ventroglútea. Las actividades fueron dirigidas por un profesor, con el apoyo de un mediador y al menos dos monitores. Las clases, compuestas en promedio por 14 profesionales, permitieron un seguimiento individualizado y una mayor interacción en los debates y las prácticas.

**Conclusión:**

Este informe contribuye a demostrar la viabilidad de la implementación de una capacitación teórico-práctica orientada a la administración de inmunobiológicos en la región ventroglútea en el contexto de la Atención Primaria en Salud, evidenciando el potencial para mejorar la práctica profesional y aumentar la seguridad en el proceso de vacunación.

## INTRODUCCIÓN

La vacunación constituye un campo complejo y en constante evolución, correspondiendo al equipo de enfermería la responsabilidad de todas las etapas del proceso^(1)^. Esta complejidad se deriva de las frecuentes actualizaciones de los calendarios de vacunación, de la incorporación de nuevos productos inmunológicos y de la ampliación de los grupos de edad contemplados, lo que exige una capacitación continua y una supervisión sistemática de los profesionales que trabajan en las salas de vacunación^(1,2,3)^.

Esta constante transformación no siempre permite que los profesionales estén preparados para comprenderla y compartirla adecuadamente, lo que refuerza la importancia de capacitaciones bien estructuradas, que combinen la teoría con la aplicación práctica de la vacunación^(4)^. Las evidencias indican que las capacitaciones estructuradas pueden mejorar significativamente las prácticas de administración de inmunobiológicos, estandarizar los procedimientos y aumentar la seguridad durante el procedimiento de vacunación^(5,6,7)^.

Aunque una atención de calidad debe basarse en conocimientos actualizados, a menudo se observa la ausencia de procesos de capacitación continua, lo que da lugar a dudas y dificultades en el día a día del trabajo, despertando sentimientos como miedo, inseguridad y recelo^(2)^. Las evidencias indican que las capacitaciones se han realizado de manera puntual y se han orientado predominantemente a la atención de demandas emergentes, lo que refuerza la necesidad de inversiones en educación continua^(8)^. Por lo tanto, se refuerza la necesidad de capacitar al equipo de enfermería para que actúe en todas las fases del proceso de vacunación.

El calendario de vacunación del Programa Nacional de Inmunizaciones de Brasil ofrece 51 productos inmunobiológicos, como vacunas, sueros e inmunoglobulinas, garantizando la protección de los usuarios de la red pública de salud a lo largo de toda la vida^(9)^. La mayoría de estos productos inmunobiológicos se administran por vía intramuscular, lo que refuerza la importancia de adoptar técnicas seguras de administración. El uso inadecuado de esta vía puede provocar complicaciones para el paciente, como abscesos, irritaciones tisulares, neuropatías y dolores, lo que hace imprescindible el rigor técnico en la definición de la región de aplicación y en la ejecución del procedimiento^(10,11)^.

Se recomiendan los músculos deltoides y vasto lateral del muslo, así como las regiones dorsoglútea y ventroglútea, para la administración intramuscular. La región dorsoglútea está indicada para la administración de determinadas inmunoglobulinas, pero se considera la última opción para los inmunobiológicos, debido al mayor riesgo de lesión del nervio ciático y a las variaciones anatómicas que pueden comprometer la correcta aplicación del inmunobiológico^(11,12,13)^. Por su parte, la región ventroglútea se indica como primera opción en la administración en adolescentes y adultos, ya que presenta facilidad de acceso y delimitación anatómica, además de no estar cerca de nervios y vasos importantes, lo que reduce el riesgo de lesiones y garantiza mayor seguridad, siendo también ventajosa en la reducción del dolor durante la administración^(11,14,15,16)^. Aunque es anatómicamente ventajosa y ampliamente recomendada, los estudios señalan su subutilización asociada al desconocimiento de la técnica, a la inseguridad profesional y a la persistencia en el uso de métodos tradicionales^(11,14,17)^.

Por lo tanto, se pone de manifiesto la necesidad de capacitar y actualizar a los profesionales de enfermería en cuanto a la administración de inmunobiológicos en la región ventroglútea, a fin de incorporarla de manera segura y eficaz en la rutina de las salas de vacunación.

En este sentido, el objetivo del estudio es relatar la experiencia de planificación y desarrollo de la capacitación para la administración de inmunobiológicos en la región ventroglútea.

## MÉTODO

### Tipo y Lugar del Estudio

Se trata de un relato de experiencia, que permite la sistematización, el análisis y la reflexión crítica sobre las prácticas desarrolladas en el contexto profesional. Este tipo de estudio permite la producción de conocimiento a partir de la experiencia de los autores, contribuyendo a la cualificación de las prácticas en salud y enfermería^(18)^. Para el análisis del rigor metodológico del informe, se utilizaron las recomendaciones de los *Standards for QUality Improvement Reporting Excellence* (SQUIRE 2.0)^(19)^.

La capacitación se llevó a cabo en septiembre de 2024, mediante la integración de los proyectos de extensión «Educar para Vacunar» y «Conociendo el Cuerpo Humano», de la Universidad Federal de São João del-Rei, y del proyecto «Vacunación en Foco», de la Universidad Estatal de Minas Gerais, en colaboración con la Secretaría Municipal de Salud de Divinópolis, municipio del estado de Minas Gerais, en la región sudeste de Brasil.

### Objetivo de la Experiencia

Perfeccionar los conocimientos teóricos y prácticos sobre la técnica de administración de productos inmunobiológicos en la región ventroglútea, promoviendo la seguridad, la calidad de la atención y la ampliación del uso de un sitio de aplicación reconocido como seguro y eficaz, pero aún infrautilizado en la práctica cotidiana de las salas de vacunación del municipio en cuestión.

### Descripción de la Experiencia

La capacitación se llevó a cabo de manera presencial con un enfoque teórico-práctico. Se condujo basándose en un enfoque problematizador, dialógico y contextualizado, respetando los contextos sociales, culturales e históricos de los participantes^(20)^. De este modo, se promovió la construcción colectiva del conocimiento, fortaleciendo el compromiso con la mejora de los servicios de salud y la valorización de las experiencias de los profesionales de enfermería que trabajan en las salas de vacunación de las unidades de Atención Primaria de Salud.

El proceso de capacitación fue cuidadosamente planificado para garantizar la eficacia del contenido y la cualificación de los participantes. En la primera reunión, se elaboró el plan de acción, con la organización de las actividades, la distribución de horarios y la definición de las funciones de cada miembro del equipo.

Se estableció que cada sesión de capacitación tendría una duración de tres horas, abarcando dos ejes principales: (1) el estudio de la anatomía de los sistemas muscular y esquelético; y (2) contenidos teórico-prácticos sobre la administración de inmunobiológicos.

Como parte de la etapa de preparación y perfeccionamiento pedagógico, el equipo encargado de impartir la capacitación participó en una capacitación con una docente del proyecto «Conociendo el cuerpo humano», cuya temática abordó la anatomía del sistema muscular y esquelético. El enfoque se centró en los puntos de referencia anatómicos, que ayudan a identificar los lugares de aplicación de los inmunobiológicos, incluyendo los músculos deltoides, vasto lateral del muslo y la región dorsoventral del glúteo (Figura 1A). La profundización en los conocimientos anatómicos fue fundamental para ampliar la integración entre la teoría y la práctica, formando a profesionales más conscientes de sus responsabilidades y comprometidos con la mejora de la calidad de la atención^(21)^.

**Figura 1:** Etapa de preparación y perfeccionamiento pedagógico del equipo – Divinópolis, MG, Brasil, 2024.

Además, con el fin de garantizar la uniformidad y la coherencia del contenido que se impartiría, los responsables de la capacitación se reunieron previamente para analizar las divergencias encontradas en la literatura científica sobre la administración de inmunobiológicos y para colaborar en la elaboración de los materiales didácticos. Este momento de alineación permitió la estandarización de la información y la adaptación de los contenidos a las prácticas más actualizadas y seguras (Figura 1B).

Cabe destacar que el equipo adoptó la técnica de localización de la región ventroglútea basada en referencias geométricas, descrita en la literatura como un método confiable para identificar el sitio de administración intramuscular^(16)^. Para ello, se utilizan puntos anatómicos palpables, como el trocánter mayor del fémur y la espina ilíaca anterosuperior, formando un triángulo imaginario a lo largo de la cresta ilíaca. Esta técnica tiene como objetivo reducir la proximidad a estructuras nerviosas y vasculares críticas, lo que puede favorecer una mayor seguridad en la administración de inmunobiológicos en comparación con otros sitios de aplicación^(12,16)^.

Este relato de experiencia refleja las percepciones de las autoras sobre la experiencia presentada y discutida a lo largo del artículo. Al no implicar ningún tipo de recolección de datos, no fue necesario someterlo al Comité de Ética en Investigación. Además, los participantes autorizaron el uso de los registros fotográficos con fines científicos, mediante consentimiento, garantizándose la anonimidad de los involucrados.

## RESULTADOS

Inicialmente, se invitó a participar a todos los profesionales de la red que trabajaban en las salas de vacunación del municipio, incluidos aquellos vinculados a las unidades con horario extendido, que desempeñan sus actividades entre las 16:00 y las 22:00 horas y que, en general, no participan en las capacitaciones convencionales, ya que estas se realizan predominantemente durante el período diurno. Para ello, se envió una carta de invitación a los responsables técnicos de cada unidad de Atención Primaria de Salud con las opciones de fechas y horarios disponibles. Estos profesionales se encargaron de coordinarse con los supervisores y de organizar la participación de los trabajadores que prestan servicios en las salas de vacunación.

De conformidad con el cronograma previamente acordado con la Secretaría Municipal de Salud de Divinópolis, se llevaron a cabo 23 capacitaciones a lo largo de dos semanas del mes de septiembre de 2024 (del 2 de septiembre al 13 de septiembre de 2024). Las actividades se llevaron a cabo en los turnos de la mañana (n = 10), la tarde (n = 10) y la noche (n = 3), con el fin de permitir la participación del mayor número posible de profesionales del municipio y de las instituciones asociadas.

Se capacitó a 278 participantes, distribuidos de la siguiente manera: profesionales de enfermería de las salas de vacunación del municipio (n = 249); profesionales del Centro de Referencia para Inmunobiológicos Especiales del municipio (n = 5); representantes de instituciones de educación superior asociadas (n = 13); y profesionales vinculados a la Secretaría Municipal de Salud de un municipio vecino, también socio de la iniciativa (n = 11).

Cada sesión de capacitación, que tuvo una duración de tres horas, se organizó en tres partes: enfoque teórico sobre anatomía, enfoque teórico sobre el proceso de administración de inmunobiológicos y enfoque práctico, que incluyó la ejecución de la técnica de localización del sitio de administración de inmunobiológicos en la región ventroglútea.

Las actividades fueron dirigidas por un profesor responsable, con el apoyo de un profesor mediador y, como mínimo, dos estudiantes monitores, todos previamente preparados para desempeñar estas funciones. En cada sesión de capacitación participaron, en promedio, 14 personas, lo que permitió un seguimiento individualizado y favoreció la interacción durante los debates y las actividades prácticas.

### Primera Etapa: Enfoque Teórico Sobre Anatomía

Esta etapa permitió mejorar los conocimientos y aclarar dudas sobre la anatomía de los músculos más utilizados en la práctica de vacunación, además de profundizar en el estudio de la región ventroglútea, eje central de la capacitación. Para ello, se utilizaron modelos anatómicos que facilitaron la visualización y la delimitación de las estructuras. En la extremidad superior, se exploró la localización del músculo deltoides; en la extremidad inferior, se exploró la identificación del vasto lateral del muslo; y en la pelvis, se exploró la observación de las referencias óseas junto con el fémur, estructuras importantes para la localización de la región ventroglútea. Además, se utilizó una pieza anatómica del cuerpo humano con estructuras removibles de los músculos glúteos (mayor, medio y menor), lo que permitió la visualización precisa de la región ventroglútea (Figura 2).

**Figura 2:** Enfoque teórico sobre anatomía – Divinópolis, MG, Brasil, 2024.

### Segunda Etapa: Enfoque Teórico Sobre el Proceso de Administración de Productos Inmunológicos

Esta etapa se llevó a cabo mediante una clase apoyada en recursos digitales, con una presentación en formato de diapositivas. Con una metodología participativa, la clase abordó temas esenciales, tales como: el concepto de vacunación; los materiales necesarios; los cuidados durante la vacunación; la selección y orientación del usuario; la evaluación de la musculatura y la definición del lugar de aplicación; las vías de administración (oral, subcutánea e intramuscular); y los cuidados con la eliminación de residuos y la higiene de las manos (Figura 3).

**Figura 3:** Enfoque teórico sobre el proceso de administración de productos inmunobiológicos – Divinópolis, MG, Brasil, 2024.

Se animó a los participantes a participar en los debates colectivos, lo que permitió aclarar dudas, compartir experiencias y reflexionar críticamente sobre el día a día en las salas de vacunación. Las discusiones abarcaron percepciones sobre las condiciones estructurales, los desafíos enfrentados en la rutina laboral y la importancia de la capacitación para la cualificación de la práctica de la atención.

Se constató que uno de los principales desafíos para la implementación de la técnica de administración de inmunobiológicos en la región ventroglútea está relacionado con la comunicación entre el equipo de salud y los usuarios del servicio. Surgieron dudas sobre los enfoques más adecuados para orientar a los usuarios y aclarar los beneficios del uso de este lugar de administración. Al tratarse de una región corporal aún poco explorada en la práctica cotidiana de los vacunadores del municipio y, para muchos, considerada «nueva», se manifestó la preocupación respecto a la posibilidad de causar extrañeza o rechazo por parte de la población.

Además, se identificó cierta inseguridad entre los profesionales de la salud al realizar la técnica, hallazgo corroborado por la literatura^(11,14,17)^. Entre los más jóvenes, la principal dificultad mencionada fue la falta de experiencia práctica con la técnica. En cambio, entre los más experimentados, se destacó la resistencia al cambio, frecuentemente asociada a la consolidación de prácticas previamente establecidas y a la familiaridad con los métodos tradicionalmente utilizados. Dicha resistencia al cambio se ve corroborada por estudios internacionales^(17,22)^. Además, un estudio evidenció la escasa elección de la región ventroglútea como primera opción para la administración intramuscular, incluso entre profesionales con conocimiento previo sobre la técnica^(23)^.

También se evidenció entre los profesionales un temor respecto a la seguridad del uso de esta región para la aplicación de la técnica. En este sentido, la evidencia corrobora la seguridad de esta región^(11,14,15,16)^. Un ensayo clínico aleatorizado doble ciego demostró que la técnica de vacunación intramuscular sin aspiración, utilizada en la administración de la vacuna contra la hepatitis A, resultó segura en cuanto a eventos adversos post-vacunales cuando se comparó con la técnica convencional con aspiración^(24)^. Además, una revisión sistemática demostró que la región presenta un menor riesgo de lesión nerviosa iatrogénica, así como una menor incidencia de efectos secundarios locales y sistémicos en diferentes grupos de edad^(25)^.

Los informes sobre la inseguridad de los profesionales y el desconocimiento por parte de la población refuerzan la necesidad de acciones educativas continuas e incentivos periódicos orientados al uso de esta región anatómica. Estas acciones deben promover no solo el dominio técnico, sino también el fortalecimiento de las competencias comunicativas de los profesionales, a fin de favorecer la adhesión del equipo y la aceptación de la población respecto a la técnica. Esta necesidad también se menciona en estudios que sugieren la realización de capacitaciones basadas en evidencia científica^(26,27)^.

En este contexto, con el fin de complementar los materiales didácticos y difundir información sobre la administración de inmunobiológicos en la región ventroglútea, basada en evidencia científica, se elaboró una infografía fundamentada en documentos oficiales del Ministerio de Salud y en literatura de anatomía (https://doi.org/10.17632/hfwkt3gznn.1)^(28)^.

El objetivo principal del recurso fue facilitar la comprensión sobre la administración de inmunobiológicos y los sitios anatómicos recomendados. Gracias a su lenguaje accesible y a su organización visual, la infografía contribuyó a la asimilación del contenido, configurándose como un recurso pedagógico relevante, posteriormente impreso en tamaño de *banner* y distribuido a todas las salas de vacunación de las unidades de Atención Primaria de Salud del municipio.

El uso de materiales educativos, como la infografía desarrollada, resultó relevante para la fijación del conocimiento, ya que las estrategias visuales y los recursos didácticos estructurados favorecen la consolidación del contenido y ayudan a la estandarización de las prácticas asistenciales^(29)^. Un estudio cuasi-experimental, realizado en Estambul, Turquía, con estudiantes de enfermería, demostró que la instrucción mediante video puede mejorar la competencia en la administración de inyecciones intramusculares en la región ventroglútea, lo que indica un impacto positivo de las estrategias educativas en la cualificación de la práctica y en la seguridad de la técnica^(30)^.

La iniciativa destaca, además, que el uso de materiales educativos de apoyo y el acompañamiento institucional constituyen elementos facilitadores para la adhesión del equipo y una mayor aceptación de la técnica por parte de la población.

### Tercera Etapa: Enfoque Práctico

En esta etapa, los participantes se organizaron en parejas para practicar la localización del sitio de administración de inmunobiológicos en la región ventroglútea, basándose en referencias geométricas^(12,16)^. La capacitación incluyó las posiciones recomendadas para niños, adolescentes, adultos y adultos mayores, utilizando modelos anatómicos y marcadores para señalar la región adecuada. A quienes manifestaron interés se les brindó la oportunidad de practicar técnicas asépticas con jeringas y agujas, sin, sin embargo, realizar la administración de líquidos. Para esta actividad, se proporcionaron jeringas de 3 ml, agujas de calibres 20 × 5,5 mm, 27 × 8 mm y 25 × 7 mm, además de alcohol, algodón y una caja colectora para materiales punzocortantes (Figura 4).

**Figura 4:** Enfoque teórico sobre el proceso de administración de productos inmunológicos – Divinópolis, MG, Brasil, 2024.

Durante una sola visita a la sala de vacunación, la persona puede recibir múltiples vacunas, lo que exige, especialmente en casos de retraso en la vacunación, la aplicación en diferentes sitios anatómicos. Así, la experiencia refuerza la importancia de la educación continua estructurada como dispositivo estratégico para la incorporación de prácticas basadas en la evidencia, especialmente cuando implican cambios en rutinas consolidadas.

Durante la práctica, los participantes fueron orientados constantemente por el profesor responsable y los monitores, recibiendo feedback individual sobre la precisión de la localización anatómica y la postura adecuada para la administración. Además, se discutieron estrategias para abordar diferentes perfiles de usuarios, promoviendo el desarrollo de competencias comunicativas y la seguridad en la ejecución de la técnica. Esta etapa permitió la consolidación de los conocimientos teóricos, la familiarización con los materiales e instrumentos utilizados, así como el fortalecimiento de la confianza de los participantes para la futura aplicación de la técnica en situaciones reales.

El uso de recursos didácticos diversificados resultó fundamental para facilitar la comprensión de las localizaciones anatómicas correctas e incentivar la asimilación del contenido, contribuyendo a la cualificación del proceso de vacunación. Además, la capacitación permitió identificar desafíos importantes, como la inseguridad entre los profesionales menos experimentados, la resistencia al cambio entre los más experimentados y la necesidad de estrategias de comunicación eficaces con los usuarios, especialmente por tratarse de una técnica aún poco conocida en la rutina de las salas de vacunación. Entre las estrategias adoptadas, se destaca la puesta a disposición de la infografía elaborada en formato de banner, disponible en las salas de vacunación del municipio, con el objetivo de apoyar la práctica profesional y favorecer una comunicación clara y estandarizada con la población.

Como limitaciones, cabe destacar que los momentos no incluyeron la evaluación de la percepción y la participación de los profesionales de la salud involucrados en la capacitación, ni investigaron los impactos de la intervención en su práctica cotidiana. Se enfatiza que las investigaciones futuras podrán avanzar en este aspecto, mediante el uso de instrumentos validados y estrategias sistemáticas de evaluación del impacto de la capacitación en la práctica profesional. Aunque no se aplicaron instrumentos formales de seguimiento o evaluación de la capacitación, posteriormente se recibieron informes verbales y registros fotográficos que evidenciaron la incorporación de la técnica en algunas unidades, los cuales, sin embargo, no fueron objeto de análisis estructurado en el marco de este informe.

Cabe destacar también que, a pesar de la relevancia del tema, se observa una escasez de estudios recientes y de informes de experiencia que aborden el uso de la región ventroglútea para la administración de inmunobiológicos, especialmente en el contexto nacional. La literatura disponible se centra, predominantemente, en aspectos técnicos de la administración intramuscular, lo que pone de manifiesto lagunas en cuanto a la implementación, la aceptación y la capacitación profesional orientadas al uso de la región ventroglútea en la práctica cotidiana de las salas de vacunación.

La Secretaría Municipal de Salud del municipio invierte continuamente en la actualización y la capacitación de sus profesionales para que estén plenamente preparados para actuar en el manejo seguro de todo el proceso de vacunación, garantizando la atención, la protección y la seguridad de la población. En un municipio de tamaño mediano, como el de este informe, que cuenta actualmente con 45 salas de vacunación en la Atención Primaria de Salud y un Centro de Referencia para Inmunobiológicos Especiales, la educación permanente sobre un tema de tanta relevancia, como la capacitación teórico-práctica en la administración de inmunobiológicos, favoreció la difusión del conocimiento y el desarrollo de habilidades de los participantes. La capacitación también permitió identificar y corregir de inmediato fallas técnicas que pueden surgir en la rutina laboral y comprometer la calidad de la atención a los usuarios. De esta manera, este proceso contribuye a la estandarización de las prácticas entre los vacunadores, con el potencial de favorecer la seguridad del paciente y la calidad del servicio prestado.

Por último, cabe destacar la importancia de consolidar la colaboración entre las instituciones educativas y los servicios de salud, estimulando el compromiso de los vacunadores con la búsqueda del conocimiento y, en consecuencia, con la búsqueda de la calidad en las técnicas de vacunación, lo que se refleja en una mayor competencia en la ejecución de las actividades y una mayor seguridad en el día a día de las salas de vacunación del municipio.

## CONCLUSIÓN

Este informe contribuye a demostrar la viabilidad de implementar una capacitación teórico-práctica orientada a la administración de inmunobiológicos en la región ventroglútea en el contexto de la Atención Primaria de Salud, evidenciando el potencial para mejorar la práctica profesional y ampliar la seguridad en el proceso de vacunación. Al sistematizar estrategias educativas que integraron un enfoque expositivo, la discusión participativa y la simulación práctica, la intervención favoreció el desarrollo de competencias técnicas y el fortalecimiento de la confianza de los profesionales para la adopción de la región ventroglútea, un sitio anatómico aún poco explorado en la rutina de las salas de vacunación.

Al ampliar el debate sobre el uso de la región ventroglútea en la administración de inmunobiológicos, el informe ofrece información útil para gestores y profesionales interesados en mejorar la seguridad asistencial y actualizar las prácticas de enfermería en el ámbito de la vacunación.

## DISPONIBILIDAD DE DATOS

El material complementario puede consultarse en: https://doi.org/10.17632/hfwkt3gznn.1.
